# Genomic sequencing of fourteen *Bacillus thuringiensis* isolates: insights into geographic variation and phylogenetic implications

**DOI:** 10.1186/s13104-023-06411-1

**Published:** 2023-07-04

**Authors:** Michael B. Blackburn, Michael E. Sparks, Ruchir Mishra, Bryony C. Bonning

**Affiliations:** 1 Invasive Insect Biocontrol and Behavior Laboratory, USDA-ARS, Henry A Wallace Beltsville Agricultural Research Center, Beltsville, MD 20705 USA; 2 Department of Entomology and Nematology, University of Florida, Gainesville, FL 32611 USA

**Keywords:** *Bacillus thuringiensis*, *kurstaki*, *toumanoffi*, *israelensis*, *pakistani*, *entomocidus*, *finitimus*, multilocus sequence type, pesticidal proteins

## Abstract

**Objective:**

This work was performed in support of a separate study investigating the activity of pesticidal proteins produced by *Bacillus thuringiensis* against the Asian citrus psyllid, *Diaphorina citri*. The fourteen *Bacillus* isolates chosen were selected from a large, geographically diverse collection that was characterized only by biochemical phenotype and morphology of the parasporal crystal, hence, for each isolate it was desired to determine the specific pesticidal proteins produced, assign each to a *Bacillus cereus* multilocus sequence type (ST), and predict their placement within the classical *Bt* serotyping system. In addition, phylogenetic distances between the isolates and *Bacillus thuringiensis* serovar type strains were determined by calculating digital DNA-DNA hybridization (dDDH) values among the isolates.

**Results:**

Based on the assembled sequence data, the isolates were found to be likely representatives of the *Bt* serovars *kurstaki* (ST 8), *pakistani* (ST 550), *toumanoffi* (ST 240), *israelensis* (ST 16), *thuringiensis* (ST 10), *entomocidus* (ST 239), and *finitimus* (ST 171). In cases where multiple isolates occurred within a predicted serovar, pesticidal protein profiles were found to be identical, despite the geographic diversity of the isolates. As expected, the dDDH values calculated for pairwise comparisons of the isolates and their apparent corresponding *Bt* serovar type strains were quite high (> 98%), however dDDH comparisons of the isolates with other serovar type strains were often surprisingly low (< 70%) and suggest unrecognized taxa within *Bt* and the *Bacillus cereus* sensu lato.

**Supplementary Information:**

The online version contains supplementary material available at 10.1186/s13104-023-06411-1.

## Introduction

*Bacillus thuringiensis* (*Bt*) is a Gram-positive spore forming bacterium that is best known for its use in the management of certain types of insects. The factors responsible for the insecticidal activity of *Bt* are typically crystalline pesticidal proteins that are produced by the bacteria upon sporulation [1], and it is these protein crystals that are the defining feature of *Bt* as a species. Here we describe the genomic sequencing and assembly of fourteen *Bt* isolates which were screened for activity against the Asian citrus psyllid (*Diaphorina citri*); the results of those assays will be presented in a separate report. These soil isolates were from an extensive collection of *Bts* from around the world that were characterized entirely on basis of biochemical phenotype and crystal morphology [2]. As the collection is largely uncharacterized genetically, isolates were chosen to represent a diversity of biochemical traits, crystal morphologies, and geographic sources (see Additional file 1). Geographically, the fourteen isolates represented seven states from within the US, as well as Norway, Nepal, Argentina, and Vietnam.

We used the genome assemblies to identify the pesticidal proteins the isolates are predicted to produce, determined their multilocus sequence types within the *Bacillus cereus* sensu lato, and predicted the placement of the isolates within the classical *Bt* serovar classification scheme. Finally, we used a whole genome alignment method to better assess the genetic distances between the isolates and the *Bt* serovars they represent.

## Materials and methods

The isolates were grown for 8 h in 20 ml of Luria broth on an orbital shaker at 30˚ C and 200 rpm. Genomic DNA was extracted from bacterial cell pellets using Qiagen’s Purgene Yeast/Bacteria Kit B according to the manufacturer’s gram-positive bacteria protocol. DNA concentrations were determined with a QuantiFluor™-ST Fluorometer (Promega, Madison, WI) using the QuantiFluor™ dsDNA System protocol.

Quality controlled DNA was sequenced using PacBio Sequel II (operated by GENEWIZ, South Plainfield, NJ, USA) and Illumina MiSeq (operated in-house at USDA-ARS Invasive Insect Biocontrol and Behavior Laboratory, Beltsville, MD, USA) instruments. Resulting sequencing reads achieved, as well as NCBI SRA accession identifiers, are presented in Additional file 2. PacBio subreads were filtered and assembled using pbcromwell v1.2.0 (contained in the SMRT Link v10.1.0.119588 software package, downloaded November 4, 2021 from https://www.pacb.com/support/software-downloads/) employing default parameters. Assembled contigs were subsequently polished using Illumina short-read data with Pilon v1.24 [3]. Two exceptions to this protocol included the case of isolate IBL02897, for which pbcromwell failed to assemble a 350 kb plasmid encoding multiple pesticidal proteins, although clear evidence for its presence existed among unassembled subreads (Additional file 3). This strain’s subreads were independently assembled using Canu v2.2 [4], the results of which contained the plasmid of interest. The Canu-assembled plasmid was then short-read polished with Pilon and manually added to the isolate’s overall genome assembly file. The second case involved IBL03111 (putative *Bt kurstaki*), for which sequencing depth plots and comparisons with another apparent *Bt kurstaki*, isolate IBL01313, indicated that pbcromwell had erroneously incorporated a ~ 330 kb pesticidal protein gene-encoding plasmid into the assembly’s main chromosome (Additional file 3). A substitute assembly was prepared by assembling IBL03111 PacBio subreads with Canu, polishing with Illumina short-read data using Pilon and comparing results with strain IBL01313 using nucmer from the MUMmer v3.23 suite [5]. Contigs lacking evident similarity to IBL01313 were culled from the revised IBL03111 assembly; these were all quite short, ranging from 8,784 to 17,316 bases, and manual inspection suggested they were likely assembly artifacts (Additional file 4). Protein coding gene prediction was performed using Prodigal v2.6.3 [6] with default parameters. Raw read data and polished assemblies are organized under NCBI BioProject PRJNA848845.

*Bacillus cereus* multilocus sequence types (ST) of isolates were determined with the system devised by Priest et al. [7], using the sequence query utility of PubMLST [8](https://pubmlst.org/organisms/bacillus-cereus), which allows sequence types to be determined from whole genome sequence queries. In this system, isolates within a ST share 100% nucleotide sequence identity across PCR amplicons of seven single copy housekeeping genes totaling ca. 2830 nucleotides.

Classically, different types of *Bt* were distinguished from one another serologically based on antisera developed primarily against flagellar proteins [9]. In this study, prediction of classical serovar status was accomplished by finding *Bt* serovar type strains with flagellin sequences that matched those predicted for our isolates. Flagellin sequences of our isolates were identified using BLASTp of Prodigal predicted proteins, and the resulting sequences used to query GenBank. Strains of *Bt* producing identical flagellin proteins were then examined for the presence of serovar type strains.

To gauge phylogenetic distances between the isolates, classical serovar type strains, and relevant species of *Bacillus*, digital DNA-DNA hybridization (dDDH) values were calculated for pairwise genomic alignments between these groups using the Type Strain Genome Server at DSMZ at https://tygs.dsmz.de/. This method predicts the results of a classical DNA-DNA hybridization experiment based entirely on genomic sequence data, with values > 70% indicating the genomes being compared belong to the same species [10].

Potential pesticidal proteins that the isolates could produce were identified by performing local BLASTp (BLAST + 2.11.0) [11] on protein sequences predicted by Prodigal, using holotype pesticidal protein sequences obtained from the Bacterial Pesticidal Protein Resource Center (https://www.bpprc.org) [12] as queries. Potential pesticidal proteins found in this way were identified using the BestMatchFinder at the Bacterial Pesticidal Protein Resource Center. Pesticidal protein nomenclature follows that of Crickmore et al. [13].

## Results and discussion

Genome assembly and gene finding results, as well as NCBI WGS accession identifiers, are presented in Table 1; contig-specific results are shown in Additional File 5. The geographic origins of the isolates, their STs, accession identifiers of their flagellin sequences, putative serovars, and pesticidal protein profiles are summarized in Table 2.

**Table 1:** Genome assembly descriptive statistics and overall predicted protein coding gene counts

Isolate	Accession	Total size	Contigs	N50	Min contig	Max contig	[G + C]	Genes
IBL00055	JAMXJQ000000000	6,242,031	7	3,561,109	66,109	3,561,109	0.3489	6,197
IBL00090	JAMXJR000000000	6,930,987	11	5,627,117	8,406	5,627,117	0.3474	6,867
IBL00144	JAMXJS000000000	6,249,769	9	3,556,735	60,266	3,556,735	0.3490	6,204
IBL00171	JAMXJT000000000	6,903,468	11	4,970,085	5,586	4,970,085	0.3476	6,798
IBL00197	JAMXJU000000000	6,234,846	19	1,772,357	5,518	3,002,624	0.3519	6,322
IBL00210	JAMXJV000000000	6,206,836	5	5,479,495	68,198	5,479,495	0.3487	6,169
IBL00427	JAMXJW000000000	6,751,842	13	4,586,432	5,591	4,586,432	0.3498	6,900
IBL00503	JAMXJX000000000	6,267,055	13	1,396,028	7,635	2,918,783	0.3509	6,409
IBL00971	JAMXJY000000000	5,565,962	5	4,360,913	7,140	4,360,913	0.3548	5,591
IBL01259	JAMXJZ000000000	6,424,460	20	1,804,914	4,127	2,501,805	0.3506	6,621
IBL01313	JAMXKA000000000	6,850,609	15	2,779,984	7,635	2,910,989	0.3489	6,962
IBL01677	JAMXKB000000000	6,571,431	8	3,572,949	7,241	3,572,949	0.3512	6,609
IBL02897	JAMXKC000000000	6,920,748	11	5,390,607	8,406	5,390,607	0.3476	6,842
IBL03111	JAMXKD020000000	6,842,748	13	1,394,664	11,671	2,918,118	0.3490	6,965

**Table 2:** Isolate information. Sample location, multilocus sequence type, GenBank accession numbers of predicted flagellar proteins, predicted classical serovar, and predicted pesticidal proteins

Isolate	Sample origin	MLST Sequence Type	Predicted flagellar proteins	Predicted serovar	Predicted pesticidal proteins
IBL00055	Wyoming, USA	550	WP_153588523.1 WP_153588522.1	*pakistani*	Cry1Ma, Cry2Ba, Mpp15Aa, Vip3Ba
IBL00144	Florida, USA	550	WP_153588523.1 WP_153588522.1	*pakistani*	Cry1Ma, Cry2Ba, Mpp15Aa, Vip3Ba
IBL00210	Maryland, USA	550	WP_153588523.1 WP_153588522.1	*pakistani*	Cry1Ma, Cry2Ba, Mpp15Aa, Vip3Ba
IBL00090	Wyoming, USA	240	WP_001222365.1 WP_001219718.1	*toumanoffi*	App4Aa, Cry1Ab, Cry1Bb, Cry1Da, Cry1Hb, Cry1Id, Cry1Ja, Cry1Nb, Cry2Ad, Vip3Af, Vpa2Ac, Vpb1Ca, Tca complex
IBL00171	Wisconsin, USA	240	WP_001222365.1 WP_001219718.1	*toumanoffi*	App4Aa, Cry1Ab, Cry1Bb, Cry1Da, Cry1Hb, Cry1Id, Cry1Ja, Cry1Nb, Cry2Ad, Vip3Af, Vpa2Ac, Vpb1Ca, Tca complex
IBL02897	Saigon, Vietnam	240	WP_001222365.1 WP_001219718.1	*toumanoffi*	App4Aa, Cry1Ab, Cry1Bb, Cry1Da, Cry1Hb, Cry1Id, Cry1Ja, Cry1Nb, Cry2Ad, Vip3Af, Vpa2Ac, Vpb1Ca, Tca complex
IBL00503	Arizona, USA	8	WP_038413434.1 WP_001222382.1 WP_038413430.1 WP_001219711.1	*kurstaki*	Cry1Aa, Cry1Ac, Cry1Ia, Cry2Aa, Cry2Ab, Vip3Aa
IBL01259	Escobar, Argentina	8	WP_038413434.1 WP_001222382.1 WP_038413430.1 WP_001219711.1	*kurstaki*	Cry1Aa, Cry1Ac, Cry1Ia, Cry2Aa, Cry2Ab, Vip3Aa
IBL01313	West Virginia, USA	8	WP_038413434.1 WP_001222382.1 WP_038413430.1 WP_001219711.1	*kurstaki*	Cry1Aa, Cry1Ac, Cry1Ia, Cry2Aa, Cry2Ab, Vip3Aa
IBL03111	New York, USA	8	WP_038413434.1 WP_001222382.1 WP_038413430.1 WP_001219711.1	*kurstaki*	Cry1Aa, Cry1Ac, Cry1Ia, Cry2Aa, Cry2Ab, Vip3Aa
IBL00197	Maryland, USA	10	WP_001219698.1 WP_001222355.1	*thuringiensis*	Cry1Aa, Cry1Ia, Cry2Aa, Cry2Ab, Vip3Aa
IBL00427	Riksgrensen, Norway	16	WP_001222366.1	*israelensis*	Cry4Aa, Cry4Ba, Cry10Aa, Cry11Aa, Cyt1Aa, Cyt1Ca, Cyt2Ba, Mpp60Aa, Mpp60Ba
IBL00971	Gokyo Ri, Nepal	171	WP_001222360.1 WP_001219701.1	*finitimus*	Cry26Aa, Cry28Aa
IBL01677	Wyoming, USA	239	WP_001219676.1 WP_001222376.1 WP_001222373.1 WP_001222377.1	*entomocidus*	Cry1Ab, Cry1Ba

The fourteen isolates were found to represent seven distinct *B*. *cereus* STs; ST 8, ST 10, ST 16, ST 171, ST 239, ST 240, and ST 550. The PubMLST database includes a list of all isolates that have been reported to share a given ST. For each of the STs listed above, the database included a single serovar type strain of a classical *Bt* serovar, including *Bt kurstaki* HD1 (ST 8), *Bt thuringiensis* BGSC 4A3 (ST 10), *Bt israelensis* BGSC 4Q1 (ST 16), *Bt finitimus* BGSC 4B2 (ST 171), *Bt entomocidus* BGSC 4I4 (ST 239), *Bt toumanoffi* BGSC 4N1 (ST 240), and *Bt pakistani* BGSC 4P1 (ST 550). Historically, antisera for *Bt* serotyping were developed against flagellar preparations, with specificity of these antisera presumably reflecting the high variability of flagellin proteins among *Bt* varieties. In the current study, the predicted flagellin sequences for all fourteen isolates perfectly matched those of the *Bt* serovar type strain corresponding to their ST in both number of proteins and sequence identity of those proteins (Table 2). Note that the number of flagellin proteins among serotype strains cited here ranged from one to four, and no single flagellin sequence was shared among different serotype strains. While it is not possible to say that an isolate belongs to a particular serovar without actually performing the serological test, it seems exceedingly likely that these isolates represent the predicted serovars.

Prior studies have demonstrated that a given serovar can exhibit variations in pesticidal protein profiles. *Bacillus thuringiensis kurstaki* HD-1 and HD-73, both ST 8, have different Cry protein profiles and plasmids [14, 15], and it is known that *Bt morrisoni* and *tenebrionis* are the same serotype, but with very different Cry profiles [16]. In this study we found a high degree of correlation between predicted pesticidal protein profile and ST or predicted serovar, despite the geographic diversity represented among the isolates (Table 2). Thus, IBL00090, IBL00171, and IBL02897, representing *Bt toumanoffi*, had extensive complements of identical pesticidal proteins encoded on contigs of nearly identical size, despite originating from Wyoming, Wisconsin, and Vietnam. Similar results were observed for IBL00055, IBL00144, and IBL00210 representing *Bt pakistani* from Wyoming, Florida, and Maryland respectively. The four apparent *Bt kurstaki* isolates were also found to have identical assortments of pesticidal proteins, although the size of the contigs on which they were encoded varied more than was observed for the *Bt pakistani* or *toumanoffi* isolates.

While these results might have been expected for *Bt kurstaki*, which has been spread around the world as a bioinsecticide, it was somewhat surprising for *Bt pakistani* and *toumanoffi*, which are presumably not widely dispersed by human activity. We did find that IBL00971, an apparent *Bt finitimus*, lacks a plasmid previously reported for *Bt finitimus* YBT-020 that encodes an additional copy of Cry26Aa [17], however the toxin profiles of these isolates were nevertheless identical.

The most surprising results generated in this study were the rather large phylogenetic distances between some of the classical *Bt* serovars. As expected, dDDH values obtained when comparing isolates with their corresponding serotype strains were very high (> 98%; see Table 3), however, comparisons with other serotype strains often resulted in values well below the 70% minimum expected of conspecific strains. The dDDH calculations obtained suggest that while *Bt kurstaki*, *pakistani*, *thuringiensis*, and *entomocidus* group with *Bacillus cereus* ATCC 14579^T^, *Bt israelensis* and *toumanoffi* did not group with *B*. *cereus* ATCC 14579^T^, *B. thuringiensis* ATCC 10792^T^, or any other type strains represented in the TYGS database and may represent an unrecognized taxon. Likewise, IBL00971 and *Bt finitimus* BGSC 4B2 appeared most closely related to the recently described *B*. *paranthracis* [18] but produced dDDH values which fell just below the critical 70% cutoff required to be considered examples of that species. A larger phylogenetic analysis based on several of the recent genomic alignment methods might be very useful for reexamining species boundaries within the *Bacillus cereus* group.

**Table 3:** Formula d4 dDDH values (%) calculated by comparing genomic sequences of the indicated isolates with those of serovar type strains of selected *Bt* serovars and selected *B. cereus* group type strains. Shaded cells indicate dDDH values exceeding 70%

	IBL00055	IBL00144	IBL00210	IBL00503	IBL01259	IBL01313	IBL03111	IBL01677	IBL00090	IBL00171	IBL02897	IBL00427	IBL00197	IBL00971
*Bt* serovar *pakistani* BGSC 4P1	100.0	100.0	100.0	83.7	83.5	82.6	82.7	71.9	63.6	63.8	63.7	64.3	66.3	44.5
*Bt* serovar *thuringiensis* BGSC 4A3	65.6	65.6	65.6	67.0	67.0	66.9	66.9	68.3	67.5	67.5	67.5	68.4	98.6	44.2
*Bt* serovar *toumanoffi* BGSC 4N1	63.8	63.8	63.7	64.1	64.1	64.3	64.3	64.6	99.8	99.8	99.8	72.9	67.9	44.1
*Bt* serovar *entomocidus* BGSC 4I4	72.1	72.1	72.0	71.9	71.8	71.9	72.0	99.7	65.7	65.4	65.7	64.6	68.1	45.2
*Bt* serovar *israelensis* BGSC 4Q1	62.8	62.8	62.8	64.1	64.0	63.9	63.9	64.8	73.7	73.9	73.7	99.6	69.4	44.1
*Bt* serovar *kurstaki* HD-1	82.6	82.6	82.6	97.9	97.8	98.3	98.2	72.0	64.5	64.3	64.5	63.6	68.9	44.5
*Bt* serovar *finitimus* BGSC 4B2	44.4	44.4	44.4	44.2	44.2	44.2	44.2	44.7	44.0	44.0	44.0	44.0	44.2	99.9
*B. cereus* ATCC 14579^T^	73.4	73.4	73.4	73.5	73.4	73.2	73.2	82.6	65.7	65.8	65.7	65.8	71.5	45.3
*B. thuringiensis* ATCC 10792^T^	65.9	65.9	65.9	67.3	67.3	67.3	67.3	69.0	67.8	67.9	67.8	68.7	98.6	44.3
*B. anthracis* ATCC 14578^T^	44.4	44.5	44.4	44.1	44.2	44.0	44.0	44.2	44.0	44.0	44.0	44.1	44.3	59.8
*B. paranthracis* MCCC 1A00395^T^	44.8	44.8	44.7	44.8	44.7	44.6	44.6	44.7	44.0	44.1	44.0	44.1	44.6	69.2

### Limitations

In this report, *Bt* serovars are predicted based on flagellin sequences of the isolates and those of the original serovar type strains, and not an actual serological test. They are only predictions.

## Electronic supplementary material

Below is the link to the electronic supplementary material.

Additional file 1.pdf. Biochemical phenotype, parasporal crystal morphology, and geographic origin of isolates used in this study. Biochemical traits, crystal morphologies, and geographic sources of isolates.

Additional file 2.pdf. Isolate-specific read sequencing volumes achieved for PacBio Sequel II and Illumina MiSeq instruments. Sequencing volumes achieved for each isolate and NCBI SRA accession identifiers.

Additional file 3.pdf. Errors observed in pbcromwell-based assemblies for IBL03111 and IBL02897. Describes assembly errors that prompted correcting pbcromwell-only assembly results with Canu-generated results.

Additional file 4.txt. Contigs culled from Canu-based IBL03111 assembly. Contigs from IBL03111 lacking similarity to IBL01313 and that may have been assembly artifacts.

Additional file 5.pdf. Contig accession numbers, sizes, and number of predicted proteins. Provides disaggregated, contig-specific assembly and gene finding results.

## Data Availability

Raw read data and polished assemblies are organized under NCBI BioProject PRJNA848845. *Bacillus* isolates used in this study are available for research purposes subject to export restrictions.

## Abbreviations

App: Alpha-helical pesticidal protein
BLASTp: Basic local alignment search tool
*Bt*: *Bacillus thuringiensis*
Cry: Crystal protein
Cyt: Cytolytic protein
dDDH: Digital DNA-DNA hybridization
DSMZ: Deutsche Sammlung von Mikroorganismen und Zellkulturen
Mpp: Mtx2-related pesticidal protein
PCR: Polymerase chain reaction
ST: Sequence type
Tca: Toxin complex a
TYGS: Type strain genome server
Vip: Vegetative insecticidal protein
Vpa: Active component of the Vpa/Vpb binary pesticidal protein ​​​​​​​
Vpb: Binding component of the Vpa/Vpb binary pesticidal protein ​​​​​​​
