# Supplementary material for: Genomic sequencing of fourteen Bacillus thuringiensis isolates: insights into geographic variation and phylogenetic implications
Source: BMC Res Notes. 2023 Jul 4;16:134. doi: 10.1186/s13104-023-06411-1 (PMC10318680; doi:10.1186/s13104-023-06411-1)
Supplement: Supplementary file 1 — Additional file 1.pdf. Biochemical phenotype, parasporal crystal morphology, and geographic origin of isolates used in this study. Biochemical traits, crystal morphologies, and geographic sources of isolates. [file 13104_2023_6411_MOESM1_ESM.pdf]

Biochemical phenotype, parasporal crystal morphology, and geographic origin of isolates used in this study.

Isolate	Phenotype	Crystal type	Country	STATE
IBL00055	TLUA	pyr	USA	WY
IBL00090	TLUA	bp	USA	WY
IBL00144	TLUA	pyr	USA	FL
IBL00171	TLU	bp	USA	WI
IBL00197	TLA	bp/cu	USA	MD
IBL00210	T	am	USA	MD
IBL00427	TL	cu	Norway	
IBL00503	TA	bp	USA	AZ
IBL00971	LSAE	attached/pyr	Nepal	
IBL01259	TL	bp/rhom	Argentina	
IBL01313	TLUA	bp	USA	WV
IBL01677	T	bp	USA	WY
IBL02897	TL	bp	Vietnam	
IBL03111	TLAE	bp/cu	USA	NY

Phenotype abbreviations:

T = amylase production, L = lecithinase production, S = acid from sucrose, U = urease production,

A = acid from salicin, E = esculin hydrolysis

Crystal type abbreviations:

am = amorphous, bp = bipyramidal, cu = cubic, pyr = pyramidal, rhom = rhombic
