# Supplementary material for: Genomic sequencing of fourteen Bacillus thuringiensis isolates: insights into geographic variation and phylogenetic implications
Source: BMC Res Notes. 2023 Jul 4;16:134. doi: 10.1186/s13104-023-06411-1 (PMC10318680; doi:10.1186/s13104-023-06411-1)
Supplement: Supplementary file 2 — Additional file 2.pdf. Isolate-specific read sequencing volumes achieved for PacBio Sequel II and Illumina MiSeq instruments. Sequencing volumes achieved for each isolate and NCBI SRA accession identifiers. [file 13104_2023_6411_MOESM2_ESM.pdf]

Isolate-specific read sequencing volumes achieved for PacBio Sequel II and Illumina MiSeq instruments.

Isolate	PacBio (Sequel II, Continuous Long Reads)					Illumina (MiSeq, paired-end)		
	Accession	Raw subreads	Raw subread bases	Filtered subreads	Filtered subread bases	Accession	Read pairs	Bases
IBL00055	SRR19749281	1,756,938	10,381,144,678	81,935	600,002,013	SRR19736598	1,004,643	290,814,130
IBL00090	SRR19749277	2,785,504	15,231,936,962	86,398	600,004,347	SRR19736594	612,228	368,561,256
IBL00144	SRR19749279	1,962,180	12,011,060,049	78,334	600,000,550	SRR19736596	1,481,461	437,527,792
IBL00171	SRR19749276	2,266,517	7,624,856,491	112,275	474,348,136	SRR19736593	942,867	406,604,214
IBL00197	SRR19749285	2,268,087	12,284,554,674	86,977	600,004,629	SRR19736602	1,331,525	392,949,738
IBL00210	SRR19749290	2,426,606	14,115,514,045	82,723	600,002,149	SRR19736607	1,303,922	387,757,447
IBL00427	SRR19749288	2,391,454	12,875,337,253	85,822	600,006,213	SRR19736605	216,554	63,405,210
IBL00503	SRR19749289	734,676	4,747,081,496	71,018	560,741,113	SRR19736606	877,546	405,989,794
IBL00971	SRR19749283	1,859,372	6,144,904,424	90,850	375,225,253	SRR19736600	2,593,877	1,325,369,060
IBL01313	SRR19749286	1,731,836	10,439,342,174	80,831	600,006,587	SRR19736603	203,254	60,041,105
IBL01259	SRR19749284	1,857,817	11,316,413,938	79,441	600,005,400	SRR19736601	1,255,878	371,206,292
IBL01677	SRR19749280	1,408,555	8,325,334,540	80,925	600,001,704	SRR19736597	203,431	60,187,278
IBL02897	SRR19749278	2,295,224	10,109,881,706	107,877	600,002,392	SRR19736595	157,426	45,714,034
IBL03111	SRR19749287	2,663,841	16,483,540,010	78,515	600,006,481	SRR19736604	1,506,948	702,667,138
