# Supplementary material for: Genomic sequencing of fourteen Bacillus thuringiensis isolates: insights into geographic variation and phylogenetic implications
Source: BMC Res Notes. 2023 Jul 4;16:134. doi: 10.1186/s13104-023-06411-1 (PMC10318680; doi:10.1186/s13104-023-06411-1)
Supplement: Supplementary file 3 — Additional file 3.pdf. Errors observed in pbcromwell-based assemblies for IBL03111 and IBL02897. Describes assembly errors that prompted correcting pbcromwell-only assembly results with Canu-generated results. [file 13104_2023_6411_MOESM3_ESM.pdf]

Additional File 3

• IBL03111 - improper integration of plasmid sequence into chromosome by pbcromwell

Supplementary Figures S1, S2 and S3 depict alignments of Cry toxin-bearing plasmids from *Bt kurstaki* strains IBL01313, IBL01259 and IBL00503, respectively, against the pbcromwell-based assembly of the IBL03111 chromosome using the Artemis Comparison Tool [1], utilizing its integrated megaBLAST [2] and MUMmer [3] aligners with default parameters. (megaBLAST is generally less stringent in identifying matches than is MUMmer.) Supplementary Figure S4 shows read depth of IBL03111's Illumina short reads (see Table 1 of the main text for relevant accessions) aligned to the full length of the pbcromwell-assembled IBL03111 chromosome, using Bowtie 2 [4], and Supplementary Figure S5 shows the same although zoomed in on the first one million bases of the chromosome. Read pileup data were visualized using Integrative Genomics Viewer v2.15.2 [5].

Figures S1, S2 and S3 demonstrate that pbcromwell v1.2.0 (contained in the SMRT Link v10.1.0.119588 software package, downloaded November 4, 2021 from https://www.pacb.com/support/software-downloads/) erroneously incorporated the IBL03111 Cry toxin-bearing plasmid into the strain's chromosome, partially in the first 50Kb of the assembly and also into the ~400-700Kb range. Figures S4 and S5 demonstrate a higher read mapping depth in these segments of the assembly, indicating origination from elements with a higher copy number than the remainder of the overall contig and implying an autonomously replicating extrachromosomal element rather than a chromosome per se.

Why pbcromwell successfully assembled the other *kurstaki* strains' long reads into distinct chromosome and plasmid entities, yet failed to do so for IBL03111 is not clear. As described in the main manuscript text, an alternate assembly was prepared using the Canu assembler [6] and this result did appropriately partition these data into chromosome and plasmid. Canu was initially used as an assembler in this study, although its results were generally more fragmentary than those achieved using pbcromwell in terms of contig count and assembly N50 (results not shown, although these can be readily reproduced by interested parties using the publicly available read sets described in this study).

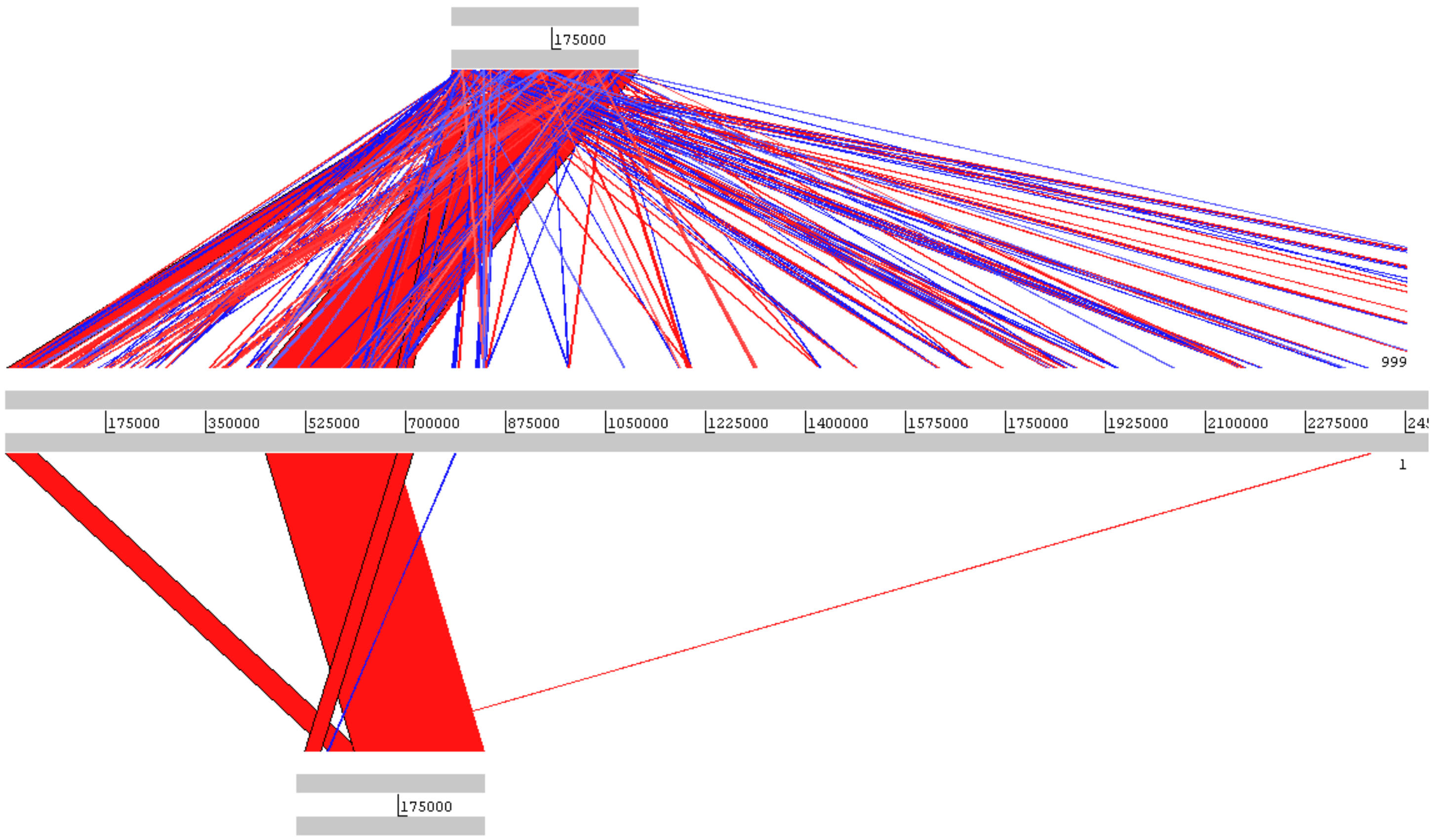

Figure S1. Alignment of *Bt kurstaki* strain IBL01313's Cry toxin-bearing plasmid against the pbcromwell-based assembly of the IBL03111 chromosome. The top- and bottom-most sequences correspond to the IBL01313 plasmid, and the middle to the IBL03111 chromosome: the upper bank of matches represent megaBLAST matches and the lower bank, MUMmer matches. Alignments presented in red occur on the forward strand, and blue on the reverse.

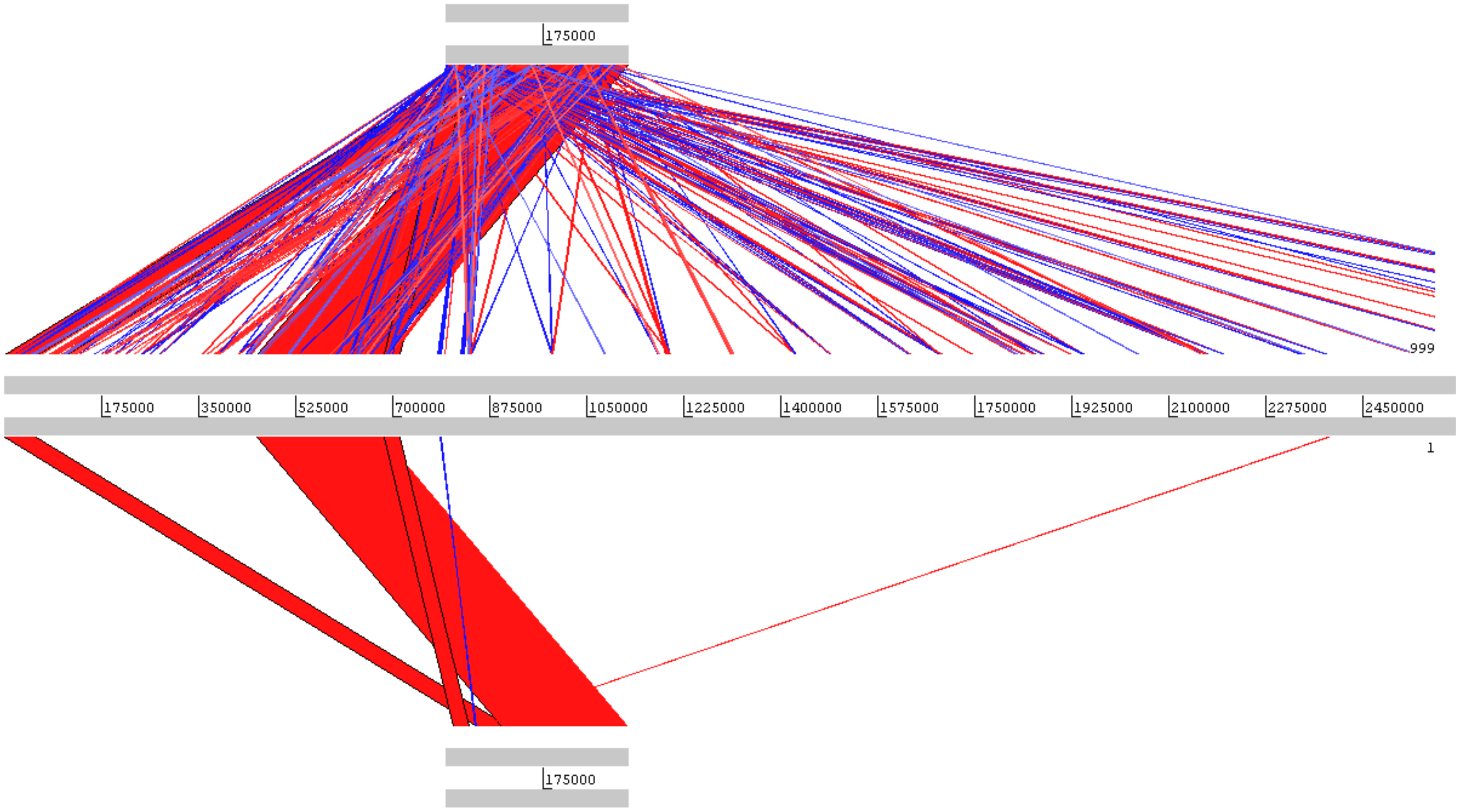

Figure S2. As presented in Figure S1, though using *Bt kurstaki* strain IBL01259's Cry toxin-bearing plasmid as a query.

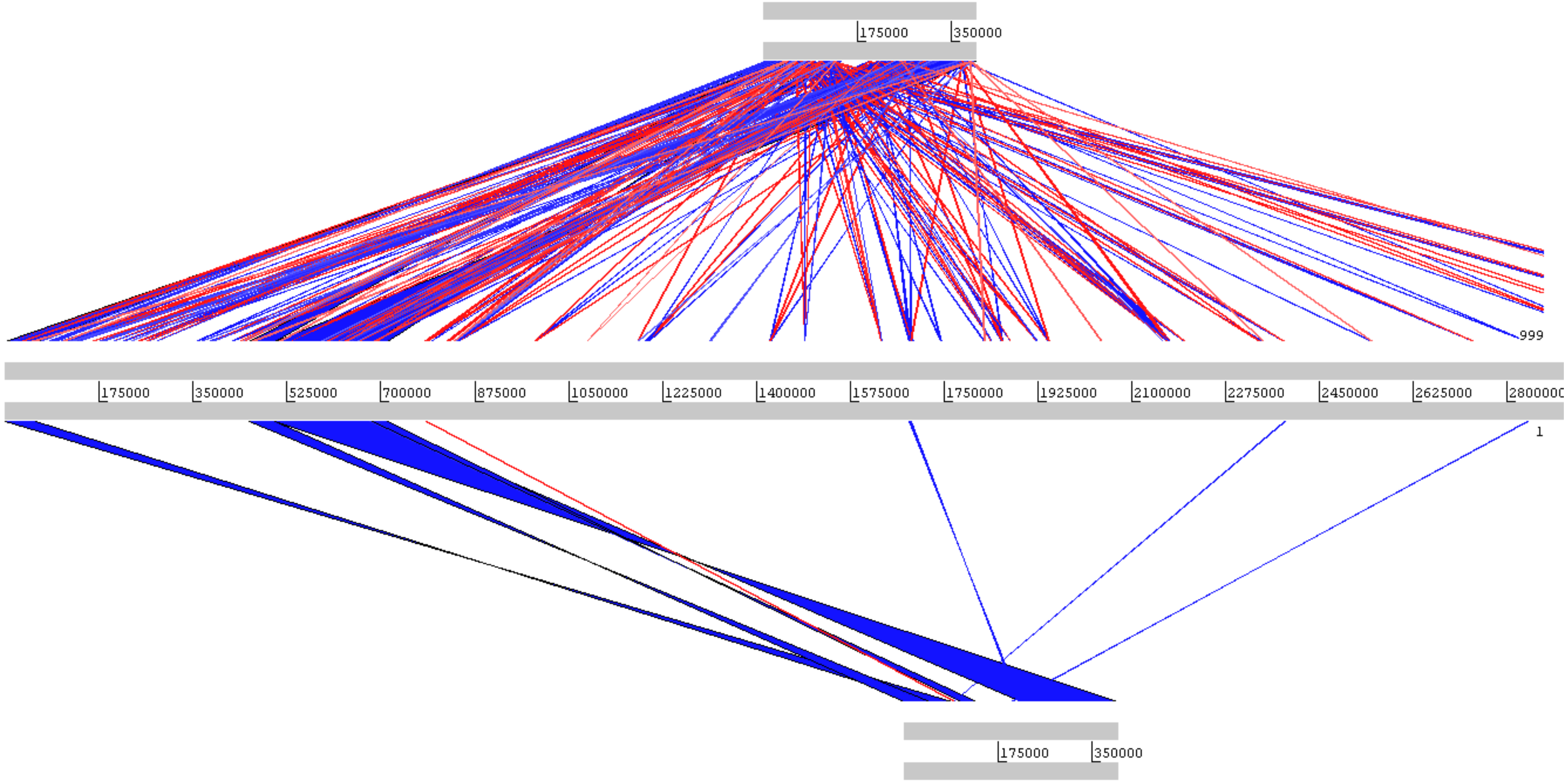

Figure S3. As presented in Figure S1, though using *Bt kurstaki* strain IBL00503's Cry toxin-bearing plasmid as a query.

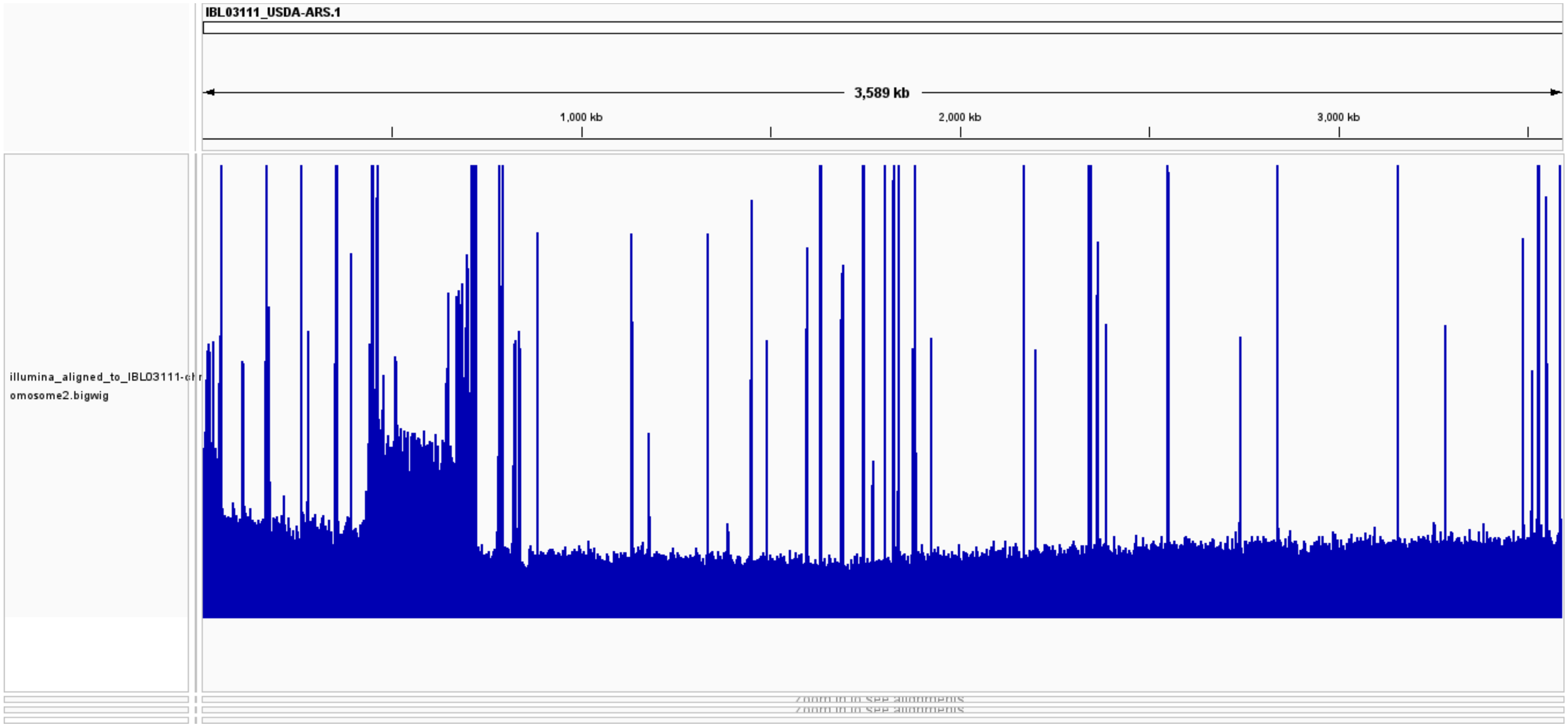

Figure S4. Read depth of short read pileup against full length of pbcromwell-assembled IBL03111 chromosome.

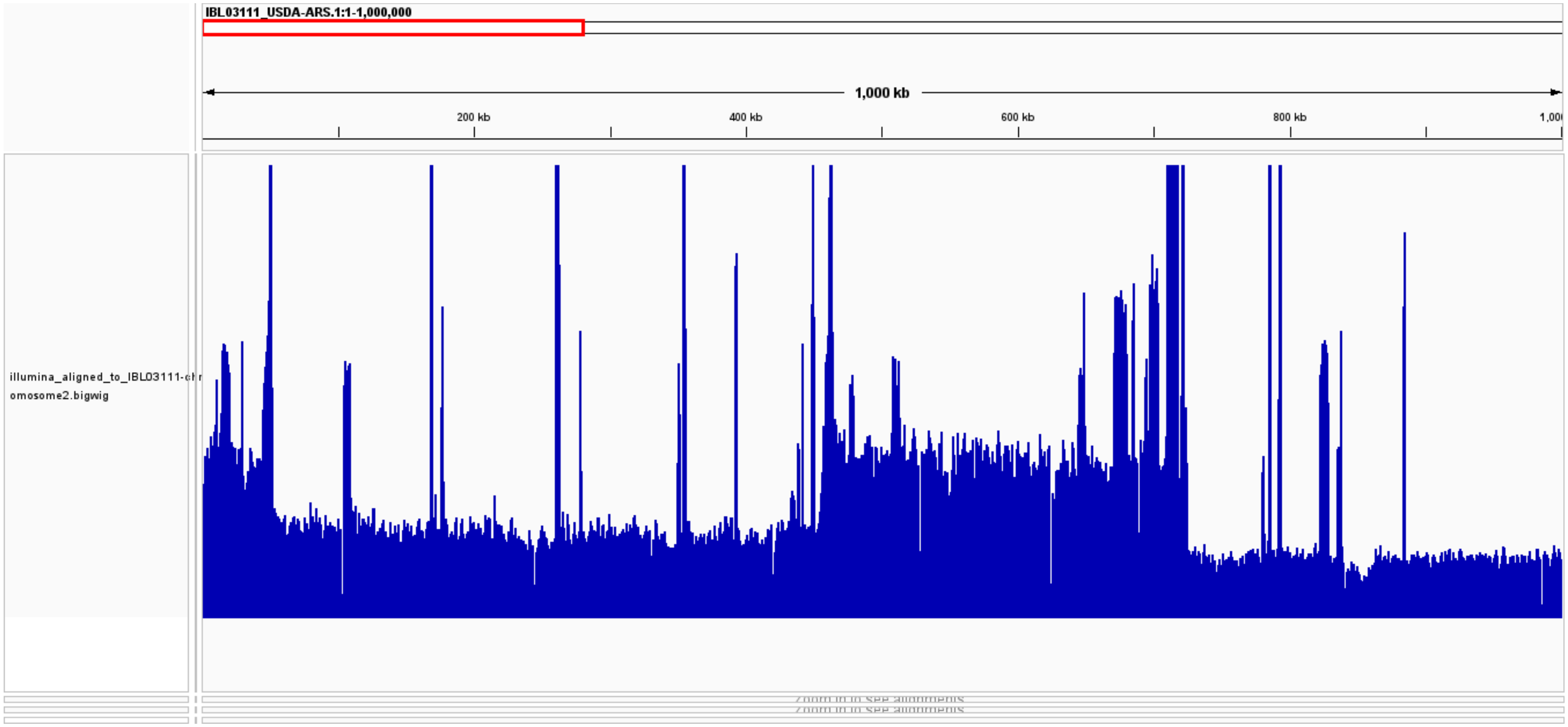

Figure S5. Read depth of short read pileup against bases 1 through 1,000,000 of pbcromwell-assembled IBL03111 chromosome.

• IBL02897 – failure to assemble plasmid sequence by pbcromwell

The pbcromwell-based IBL02897 assembly overall lacked expected plasmid sequence results encoding *Bt* toxin proteins. A provisional assembly utilizing only Illumina short reads was generated to determine whether those plasmid-borne toxin genes may be present in that data source. Quality trimming of paired-end Illumina MiSeq reads was performed using Sickle [7]. Adapter trimming and read depth normalization were performed using the BBDuk and BBNorm utilities from BBTools, respectively [8]. Processed reads were assembled into contigs using Velvet (v1.2.10) [9], with its 'k' value set to 31, and protein coding gene finding on the assembled results was done using Prodigal [10]. Twenty-seven expected toxin protein homologs were indeed found among these results and used as tBLASTn [11] queries against unassembled PacBio long reads, which indeed recovered hits and indicated their presence among long read data, also.

Querying the Canu-based IBL02897 assembly with these toxin protein sequences demonstrated that it comprised an apparent plasmid sequence in the expected size range (i.e., 350-400Kb) harboring these toxin genes. Upon short read polishing as described in the main methods, the plasmid's length was 350,358bp. It was manually added to the pbcromwell-based results. As above, determining why pbcromwell failed to correctly assemble these particular read data whereas Canu did, is beyond the scope of this investigation.

References

1. Carver TJ, Rutherford KM, Berriman M, Rajandream M-A, Barrell BG, Parkhill J. ACT: the Artemis Comparison Tool. Bioinformatics. 2005;21:3422–3.

2. Chen Y, Ye W, Zhang Y, Xu Y. High speed BLASTN: an accelerated MegaBLAST search tool. Nucleic Acids Res. 2015;43:7762–8.

3. Kurtz S, Phillippy A, Delcher AL, Smoot M, Shumway M, Antonescu C, et al. Versatile and open software for comparing large genomes. Genome Biol. 2004;5:R12.

4. Langmead B, Salzberg SL. Fast gapped-read alignment with Bowtie 2. Nat Meth. 2012;9:357–9.

5. Robinson JT, Thorvaldsdóttir H, Winckler W, Guttman M, Lander ES, Getz G, et al. Integrative genomics viewer. Nat Biotechnol. 2011;29:24–6.

6. Koren S, Walenz BP, Berlin K, Miller JR, Bergman NH, Phillippy AM. Canu: scalable and accurate long-read assembly via adaptive k-mer weighting and repeat separation. Genome Res. 2017;27:722–36.

7. Joshi NA, Fass JN. Sickle: A sliding-window, adaptive, quality-based trimming tool for FastQ files. 2011.

8. Bushnell B. The BBTools suite. 2016.

9. Zerbino DR, Birney E. Velvet: algorithms for de novo short read assembly using de Bruijn graphs. Genome Res. 2008;18:821–9.

10. Hyatt D, Chen G-L, Locascio PF, Land ML, Larimer FW, Hauser LJ. Prodigal: prokaryotic gene recognition and translation initiation site identification. BMC Bioinformatics. 2010;11:119.

11. Gertz EM, Yu Y-K, Agarwala R, Schäffer AA, Altschul SF. Composition-based statistics and translated nucleotide searches: Improving the TBLASTN module of BLAST. BMC Biol. 2006;4:41.
