# Supplementary material for: Genomic sequencing of fourteen Bacillus thuringiensis isolates: insights into geographic variation and phylogenetic implications
Source: BMC Res Notes. 2023 Jul 4;16:134. doi: 10.1186/s13104-023-06411-1 (PMC10318680; doi:10.1186/s13104-023-06411-1)
Supplement: Supplementary file 5 — Additional file 5.pdf. Contig accession numbers, sizes, and number of predicted proteins. Provides disaggregated, contig-specific assembly and gene finding results. [file 13104_2023_6411_MOESM5_ESM.pdf]

Additional File 5.

Genomic sequencing of fourteen *Bacillus thuringiensis* isolates: Insights into geographic variation and phylogenetic implications.

Contig Accession Numbers, Sizes, and Number of Predicted Proteins

Contigs encoding pesticidal proteins shaded blue.

IBL00055 *Bacillus thuringiensis pakistani*

Accession	Length	Proteins
JAMXJQ010000001.1	1,887,472	1,858
JAMXJQ010000002.1	3,561,109	3,354
JAMXJQ010000003.1	250,198	214
JAMXJQ010000004.1	334,762	252
JAMXJQ010000005.1	74,183	90
JAMXJQ010000006.1	68,198	82
JAMXJQ010000007.1	66,109	66

IBL00144 *Bacillus thuringiensis pakistani*

Accession	Length	Proteins
JAMXJS010000001.1	334,762	253
JAMXJS010000002.1	3,556,735	3,355
JAMXJS010000003.1	1,682,677	1,683
JAMXJS010000004.1	250,198	214
JAMXJS010000005.1	135,763	102
JAMXJS010000006.1	86,987	83
JAMXJS010000007.1	74,183	89
JAMXJS010000008.1	68,198	83
JAMXJS010000009.1	60,266	69

IBL00210 *Bacillus thuringiensis pakistani*

Accession	Length	Proteins
JAMXJV010000001.1	5,479,495	5,264
JAMXJV010000002.1	250,198	214
JAMXJV010000003.1	334,762	253
JAMXJV010000004.1	74,183	89
JAMXJV010000005.1	68,198	83

IBL00090 *Bacillus thuringiensis toumanoffi*

Accession	Length	Proteins
JAMXJR010000001.1	5,627,117	5,465
JAMXJR010000002.1	347,417	242
JAMXJR010000003.1	537,384	387
JAMXJR010000004.1	220,468	173
JAMXJR010000005.1	87,640	100
JAMXJR010000006.1	46,634	68

IBL00090 *Bacillus thuringiensis toumanoffi* (continued)

Accession	Length	Proteins
JAMXJR010000007.1	16,410	10
JAMXJR010000008.1	15,999	14
JAMXJR010000009.1	12,371	21
JAMXJR010000010.1	11,141	11
JAMXJR010000011.1	8,406	7

IBL00171 *Bacillus thuringiensis toumanoffi*

Accession	Length	Proteins
JAMXJT010000001.1	4,970,085	4,898
JAMXJT010000002.1	381,227	279
JAMXJT010000003.1	347,412	242
JAMXJT010000004.1	675,360	572
JAMXJT010000005.1	236,493	187
JAMXJT010000006.1	169,474	118
JAMXJT010000007.1	85,914	100
JAMXJT010000008.1	12,371	21
JAMXJT010000009.1	11,141	11
JAMXJT010000010.1	8,405	8
JAMXJT010000011.1	5,586	2

IBL02897 *Bacillus thuringiensis toumanoffi*

Accession	Length	Proteins
JAMXKC010000001.1	5,390,607	5,249
JAMXKC010000002.1	537,313	393
JAMXKC010000003.1	247,115	232
JAMXKC010000004.1	213,164	161
JAMXKC010000005.1	87,640	102
JAMXKC010000006.1	46,634	66
JAMXKC010000007.1	15,999	15
JAMXKC010000008.1	12,371	21
JAMXKC010000009.1	11,141	12
JAMXKC010000010.1	8,406	8
JAMXKC010000011.1	350,358	244

IBL00503 *Bacillus thuringiensis kurstaki*

Accession	Length	Proteins
JAMXJX010000001.1	2,918,783	2,805
JAMXJX010000002.1	1,396,028	1,341
JAMXJX010000003.1	477,999	478
JAMXJX010000004.1	82,373	82
JAMXJX010000005.1	61,908	75
JAMXJX010000006.1	46,634	68
JAMXJX010000007.1	15,160	24
JAMXJX010000008.1	14,889	23
JAMXJX010000009.1	8,514	10
JAMXJX010000010.1	8,279	10
JAMXJX010000011.1	7,635	8
JAMXJX010000012.1	832,039	853
JAMXJX010000013.1	396,814	334

IBL01259 *Bacillus thuringiensis kurstaki*

Accession	Length	Proteins
JAMXJZ010000001.1	2,501,805	2,357
JAMXJZ010000002.1	82,304	86
JAMXJZ010000003.1	55,962	53
JAMXJZ010000004.1	55,034	77
JAMXJZ010000005.1	46,634	68
JAMXJZ010000006.1	42,895	54
JAMXJZ010000007.1	26,221	34
JAMXJZ010000008.1	20,402	26
JAMXJZ010000009.1	15,646	29
JAMXJZ010000010.1	15,182	24
JAMXJZ010000011.1	14,889	23
JAMXJZ010000012.1	11,925	19
JAMXJZ010000013.1	8,279	11
JAMXJZ010000014.1	7,635	9
JAMXJZ010000015.1	4,127	5
JAMXJZ010000016.1	829,194	849
JAMXJZ010000017.1	1,804,914	1,759
JAMXJZ010000018.1	484,600	486
JAMXJZ010000019.1	314,283	241
JAMXJZ010000020.1	82,529	93

IBL01313 *Bacillus thuringiensis kurstaki*

Accession	Length	Proteins
JAMXKA010000001.1	2,779,984	2,762
JAMXKA010000002.1	2,910,989	2,787
JAMXKA010000003.1	412,003	313
JAMXKA010000004.1	326,083	251
JAMXKA010000005.1	185,778	197
JAMXKA010000006.1	80,684	76
JAMXKA010000007.1	46,634	68
JAMXKA010000008.1	24,002	18
JAMXKA010000009.1	17,084	9
JAMXKA010000010.1	14,889	22
JAMXKA010000011.1	14,854	27
JAMXKA010000012.1	13,198	11
JAMXKA010000013.1	8,513	8
JAMXKA010000014.1	8,279	9
JAMXKA010000015.1	7,635	9

IBL03111 *Bacillus thuringiensis kurstaki*

Accession	Length	Proteins
JAMXKD020000001.1	410,631	320
JAMXKD020000002.1	1,349,424	1,385
JAMXKD020000003.1	1,394,664	1,342
JAMXKD020000004.1	89,913	96
JAMXKD020000005.1	26,399	42
JAMXKD020000006.1	14,856	27
JAMXKD020000007.1	11,671	18
JAMXKD020000008.1	2,918,118	2,805
JAMXKD020000009.1	52,240	71
JAMXKD020000010.1	328,719	251
JAMXKD020000011.1	93,179	105
JAMXKD020000012.1	89,495	93
JAMXKD020000013.1	63,439	97

IBL00197 *Bacillus thuringiensis thuringiensis*

Accession	Length	Proteins
JAMXJU010000001.1	3,002,624	2,966
JAMXJU010000002.1	113,059	114

IBL00197 *Bacillus thuringiensis thuringiensis* (continued)

Accession	Length	Proteins
JAMXJU010000003.1	72,075	85
JAMXJU010000004.1	63,994	67
JAMXJU010000005.1	63,822	69
JAMXJU010000006.1	57,883	75
JAMXJU010000007.1	28,415	30
JAMXJU010000008.1	15,162	15
JAMXJU010000009.1	14,861	23
JAMXJU010000010.1	14,460	11
JAMXJU010000011.1	13,235	14
JAMXJU010000012.1	8,513	10
JAMXJU010000013.1	8,252	8
JAMXJU010000014.1	6,880	7
JAMXJU010000015.1	5,518	4
JAMXJU010000016.1	1,772,357	1,777
JAMXJU010000017.1	460,705	400
JAMXJU010000018.1	263,288	234
JAMXJU010000019.1	249,743	195

IBL00427 *Bacillus thuringiensis israelensis*

Accession	Length	Proteins
JAMXJW010000001.1	4,586,432	4,408
JAMXJW010000002.1	826,524	857
JAMXJW010000003.1	359,560	296
JAMXJW010000004.1	349,601	453
JAMXJW010000005.1	235,425	254
JAMXJW010000006.1	127,922	97
JAMXJW010000007.1	107,764	99
JAMXJW010000008.1	60,397	68
JAMXJW010000009.1	57,019	61
JAMXJW010000010.1	14,909	28
JAMXJW010000011.1	13,437	7
JAMXJW010000012.1	7,261	3
JAMXJW010000013.1	5,591	4

IBL00971 *Bacillus thuringiensis finitimus*

Accession	Length	Proteins
JAMXJY010000001.1	4,360,913	4,321
JAMXJY010000002.1	978,186	922
JAMXJY010000003.1	190,696	168
JAMXJY010000004.1	29,027	12
JAMXJY010000005.1	7,140	2

IBL01677 *Bacillus thuringiensis entomocidus*

Accession	Length	Proteins
JAMXKB010000001.1	3,572,949	3,438
JAMXKB010000002.1	1,573,046	1,432
JAMXKB010000003.1	740,622	759
JAMXKB010000004.1	463,560	359
JAMXKB010000005.1	82,404	95
JAMXKB010000006.1	79,992	68
JAMXKB010000007.1	51,617	56
JAMXKB010000008.1	7,241	2
